# The role of Syk signaling in antifungal innate immunity of human corneal epithelial cells

**DOI:** 10.1186/s12886-015-0041-z

**Published:** 2015-06-03

**Authors:** Ying Liu, Guiqiu Zhao, Jing Lin, Cui Li, Qing Li, Chengye Che, Qian Wang, Liting Hu

**Affiliations:** Department of Ophthalmology, the Affiliated Hospital of Qingdao University, Qingdao, 266003 Shandong Province China; Department of Ophthalmology, the Affiliated Hospital of Xuzhou Medical College, Xuzhou, 221000 Jiangsu Province China

**Keywords:** Syk signaling, Fungal infection, Innate immunity, Human corneal epithelial cells

## Abstract

**Background:**

Fungal keratitis is a kind of intractable and sight-threatening diseases. Spleen-tyrosine kinase (Syk) is a non-receptor tyrosine kinase, which plays an important role in the signaling pathway of the receptors. In the current study, we investigate the expression and function of Syk in human corneal epithelial cells with Aspergillus fumigatus (A. fumigatus) infection.

**Methods:**

Cultured telomerase-immortalized human corneal epithelial cells (THCEs) were treated with A. fumigatus hyphae with or without treatment of Syk inhibitors. Activation of Syk and the role of Syk in regulating inflammatory cytokines and chemokines expression were evaluated. The mRNA expression was determined by real time PCR, and protein activation was measured by western blotting.

**Results:**

Syk protein was detected in THCEs, and its activation was enhanced after treatment of A. fumigatus hyphae. Expression of inflammatory cytokines (IL-1β and IL-6) and chemokines (IL-8 and CXCL1) mRNA were significantly increased after stimulation of A. fumigatus hyphae in THCEs. Activation of Syk and expression of IL-1β, IL-6, IL-8 and CXCL1 by A. fumigatus hyphae were blocked by Syk inhibitors.

**Conclusion:**

These findings demonstrate that normal human corneal epithelial cells produce Syk, and Syk activation plays an important role in regulating A. fumigatus hyphae-induced inflammatory responses in THCEs.

## Background

Fungal keratitis is a corneal ulcer disease caused by the infection of pathogenic fungi [1]. There is a highly conservative consensus sequence named as pathogen associated molecular patterns (PAMPs) on the fungi. After the invasion of fungi, the innate immune response can immediately identify the PAMPs by pattern recognition receptors (PRRs). PRRs is the first defense line to identify and resist the pathogen infections. Then it mediates the adhesion, absorption and eradication of pathogen [2]. The PRRs that participate in the immune response of fungi mainly include Toll-like receptors (TLRs), C-type lectin receptors (CLRs) and nucleotide-binding oligomerization domain-like receptors (NLRs) [3].

Studies have shown that many CLRs, such as Dectin-1 and Mincle, resist the fungi mainly by activating the downstream Syk-CARD9-NF-κB signaling pathway [4]. A number of CLRs function on the base of signaling via association with immunoreceptor tyrosine-based activation motif (ITAM)-containing adapter proteins, while other CLRs contain ITAM-related motifs or immunoreceptor tyrosine-based inhibitory motifs (ITIMs) in their cytoplasmic tails [5]. Researchers have proved that Syk, as a potential tumor suppressor gene, was widely expressed in the hematopoietic cell lines and non-hematopoietic cell lines [6]. Its reduction or absence associates with the invasive of breast cancer [7]. Study also showed that Syk is closely related to the occurrence and development of digestive tract tumor [8]. Recent studies have revealed the importance of Syk during C. albicans infection [9]. Syk, as a non-receptor tyrosine kinase, can integrate with the protein receptors which contain ITAM motif and phosphorylated-Syk (p-Syk) can activate downstream targets. Typically, Syk can undergo auto-phosphorylation when it bound to the ITAM domain of immune response receptors [10]. Syk is the common signaling pathway of many receptors and also is the key kinase which mediates the downstream cell signaling [11]. Up to now, there is little research on whether Syk exists in human corneal epithelial cells and its function in A. fumigatus keratitis. In our research, we detected the production of p-Syk protein and the expression of inflammatory cytokines (IL-1β and IL-6) and chemokines (IL-8 and CXCL1) in the A. fumigatus hyphae infected THCEs with or without pretreatment of PRT062607 or Piceatannol, the specific Syk inhibitors. Then we preliminarily discussed the role of Syk in innate immune response of fungal infection in THCEs.

## Methods

### Materials and reagents

The THCE cells were kindly given by Xiamen eye center. Delbeccon’s modified Eagle’s medium(DMEM), Fetal Bovine Serum(FBS) and 0.25 % trypsin/0.03 % EDTA solution were purchased from Gibco (San Diego, California, USA); Dimethylsulfoxide (DMSO) was purchased from Solarbio (Beijing, China); Sabouroud medium was purchased from Babio biotech (Jinan, China); RNAiso Plus and reverse transcriptase polymerase chain reaction (RT-PCR) kits and SYBR® Premix Ex Taq™ (Tli RNaseH Plus) were purchased from TaKaRa (Dalian, China); Primary antibodies against phospho-Syk and Syk came from Cell Signaling Technology (Danvers, MA). A mouse antibody against GAPDH, Bicinchoninic Acid Assay, ECL Western Blotting Detection Reagent were from Beyotime (Shanghai, China); The secondary antibodies were from Cwbiotech (Beijing, China); Syk inhibitors, PRT062607 and Piceatannol, were purchased from Selleck Chemicals (Houston, USA); Phenylmethylsulphonylfluoride (PMSF) and radio immunoprecipitation assay (RIPA) lysis buffer were purchased from Solarbio (Beijing, China).

### Preparation of Aspergillus fumigates hyphae

The standard A. fumigatus strain was purchased from China General Microbiological Culture Collection Center (CGMCC) and grown in Sabouroud medium at 28 °C for 5–7 days. Then the conidia were inoculated to liquid medium at 37 °C for 3–4 days. We collected the hyphae after centrifugation and grinded hyphae to the size of 20 ~ 40 μm fragment. The hyphae were inactivated by treatment with 75 % ethanol overnight, washed for 3 times by sterile phosphate buffer saline solution (PBS) and adjusted the concentration to 5 × 10^6^/ml with DMEM [12].

### Culture of human corneal epithelial model

THCEs were cultured in DMEM with 10 % FBS, 0.075 % growth factor, 0.075 % insulin, 1 % penicillin G and streptomycin sulfate at 37 °C in a humidified atmosphere containing 5 % CO_2_, and the medium was replaced every 2 days. Confluent corneal epithelial cultures were switched to serum-free DMEM and treated with A. fumigatus hyphae for 15, 30, 45, 60 min in 6-well plates and 4, 8, 16 h in 12-well plates. After treatment of 4–16 h, cells were lysed for total RNA extraction and mRNA detection. After treatment of 0–60 min, cell lysate was collected for immunoassay. To investigate the function of Syk, the inhibition experiments were done. THCEs were pre-incubated with specific Syk inhibitors PRT062607 (1 and 2 μM) [13] and Piceatannol (5 and 10 μM) [14] for 30 min. Then added A. fumigatus hyphae and incubated for 45 min, 4 h and 8 h, respectively. Cells with or without pretreatment of Syk inhibitors were uesd for PCR and western blotting. Piceatannol dissolved in DMSO, preliminary experiments showed no obvious difference between the DMSO group and normal group.

### Western blotting analysis

Protein was extracted from cultured cells via RIPA lysis buffer plus 1 mM PMSF. Total protein was quantified via Bicinchoninic Acid Assay, denatured with SDS-PAGE Sample Loading Buffer at 95 °C for 5 min. Proteins (40 μg/well) were separated by 12 % sodium dodecyl sulphate–polyacrylamide gel electrophoresis (SDS-PAGE) in Tris/glycine/SDS buffer and electro-blotted onto nitrocellulose transfer membranes. After blocking by the Western blocking buffer for 2 h the membranes incubated with primary antibodies of p-Syk, Syk and GAPDH at 4 °C overnight. Then membranes were incubated with secondary antibodies for 1.5 h. All blots were detected with BeyoECL Plus.

### RNA isolation and real time RT-PCR assay

Cells were harvested and saved in −80 °C. Total RNA was isolated from cells by RNAiso plus reagent and quantified by spectrophotometry rapidly. RNA (2 μg) was used for first-strand cDNA synthesis according to the protocol for a reverse transcription system. Then cDNA (2 μl) was used for PCR in 20 μl reaction volume following the manufacturer’s instructions. Primer pairs of IL-1β, IL-6, IL-8, CXCL1 and β-actin were shown in Table 1. All reactions performed following cycling parameters: 95 °C for 30s, followed by 40 cycles of 94 °C for 5 s, 60 °C for 30s, 95 °C for 15 s and 60 °C for 30s, followed by a final stage of 95 °C for 15 s. Quantification of gene expression was analyzed by the comparative threshold cycle (CT) method and normalized by β-actin [15].

**Table 1:** Primer list used for RT qPCR and the product size

	Forward primer 5′-3′	Reverse primer 5′-3′	Product size
β-actin	TGGCACCCAGCACAATGAA	CTAAGTCATAGTCCGCCTAGAAGCA	186 bp
IL-1β	GCTGATGGCCCTAAACAGATGAA	TCCATGGCCACAACAACTGAC	140 bp
IL-6	AAGCCAGAGCTGTGCAGATGAGTA	TGTCCTGCAGCCACTGGTTC	150 bp
IL-8	TTTCAGAGACAGCAGAGCACACAA	CACACAGAGCTGCAGAAATCAGG	145 bp
CXCL1	AGGGAATTCACCCCAAGAAC	CACCAGTGAGCTTCCTCCTC	193 bp

### Statistical Analysis

All data shown are the mean ± SD from at least three independent experiments. Data analysis was done by One-way ANOVA test and further comparison in pairs was analysed by LSD test using SPSS17.0 software. Values were considered significant at p < 0.05.

## Results

### A. fumigatus hyphae stimulated the expression of inflammatory mediators in THCEs

**Fig. 1:**
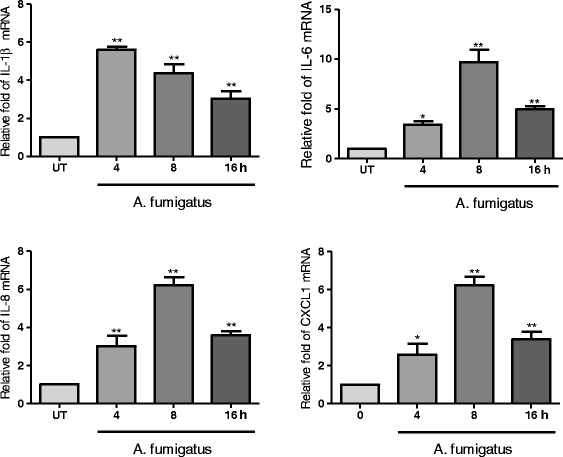
A. fumigatus hyphae induced mRNA expression of inflammatory mediators in THCEs. The mRNA expression of inflammatory cytokines (IL-1β and IL-6) and chemokines (IL-8 and CXCL1) in THCEs were evaluated at 4, 8, 16 h. IL-1β (p < 0.01, p < 0.01, p < 0.01), IL-6 (p < 0.05, p < 0.01, p < 0.01), IL-8 (p < 0.01, p < 0.01, p < 0.01) and CXCL1 (p < 0.05, p < 0.01, p < 0.01) mRNA levels were elevated after A. fumigatus hyphae stimulation of 4, 8, 16 h separately compared with untreated normal THCEs. **p < 0.01, *p < 0.05

To explore inflammatory response of THCEs stimulated by A. fumigatus hyphae (5 × 10^6^/mL), the mRNA expression of inflammatory cytokines (IL-1β and IL-6) and chemokines (IL-8 and CXCL1) in THCEs were evaluated by qPCR at 4, 8, 16 h. IL-1β (p < 0.01, p < 0.01, p < 0.01), IL-6 (p < 0.05, p < 0.01, p < 0.01), IL-8 (p < 0.01, p < 0.01, p < 0.01) and CXCL1 (p < 0.05, p < 0.01, p < 0.01) mRNA levels were elevated after the A. fumigatus hyphae stimulation of 4, 8, 16 h compared with untreated normal THCEs. For IL-6, IL-8 and CXCL1, the mRNA expression reached peak after treatment of A. fumigatus hyphae for 8 h, while the expression of IL-1β mRNA reached its peak at 4 h (Fig. 1). These results showed the expression of inflammatory cytokines (IL-1β and IL-6) and chemokines (IL-8 and CXCL1) in THCEs increased after the stimulation of A. fumigatus hyphae.

### Activation of Syk in THCEs stimulated by A. fumigatus

**Fig. 2:**
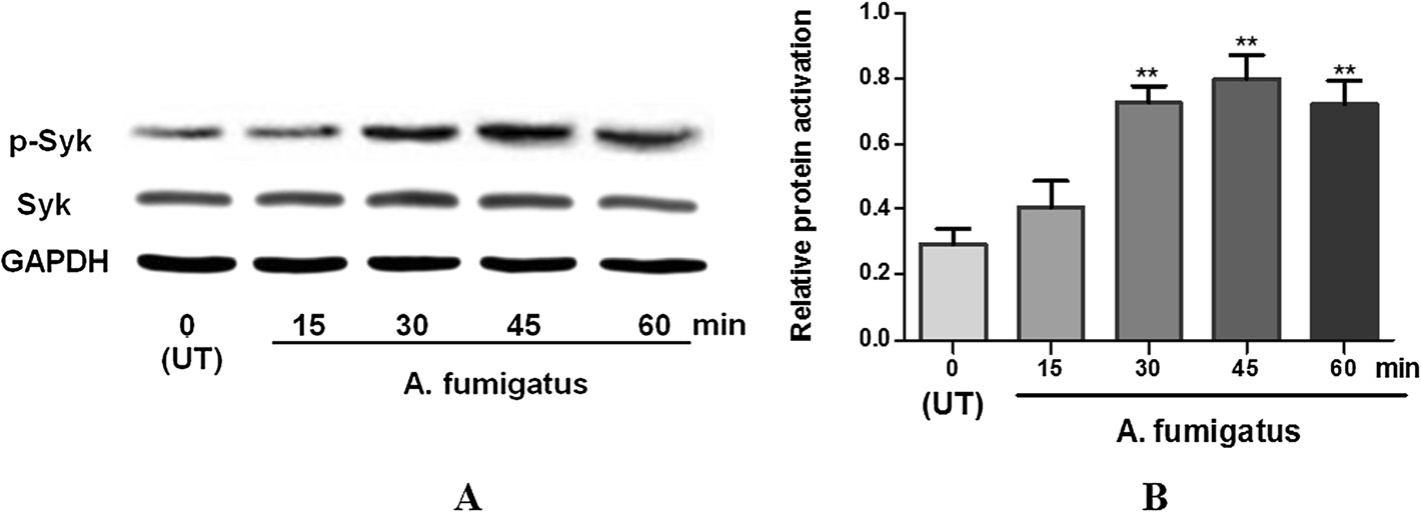
Syk and p-Syk in THCEs before or after A. fumigatus hyphae stimulation. There was no significant difference between infected cells and normal control cells for Syk protein expression. p-Syk was elevated in the infected THCEs compared with the control at 30, 45, and 60 min **a**. p-Syk levels were upregulated after 30 min incubation with A. fumigatus hyphae and sustained for 45 and 60 min **b** (p < 0.01, p < 0.01, p < 0.01 separately). **p < 0.01, *p < 0.05 compared with normal control

To investigate the activation of Syk after A. fumigatus hyphae treatment, THCEs were incubated with A. fumigatus hyphae (5 × 10^6^/mL) for 15, 30, 45, and 60 min, followed by western blotting. There was no significant difference between infected cells and normal control cells for Syk protein expression. However, p-Syk was elevated in the infected THCEs compared with the control at 30, 45, and 60 min (Fig. 2a). p-Syk levels were upregulated after 30 min incubation with A. fumigatus hyphae and sustained for 45 and 60 min (Fig. 2b, p < 0.01, p < 0.01, p < 0.01 separately). These findings indicated the presence of Syk in THCEs and its activation by A. fumigatus hyphae.

### A. fumigatus hyphae stimulated inflammatory mediators via Syk signaling in THCEs

**Fig. 3:**
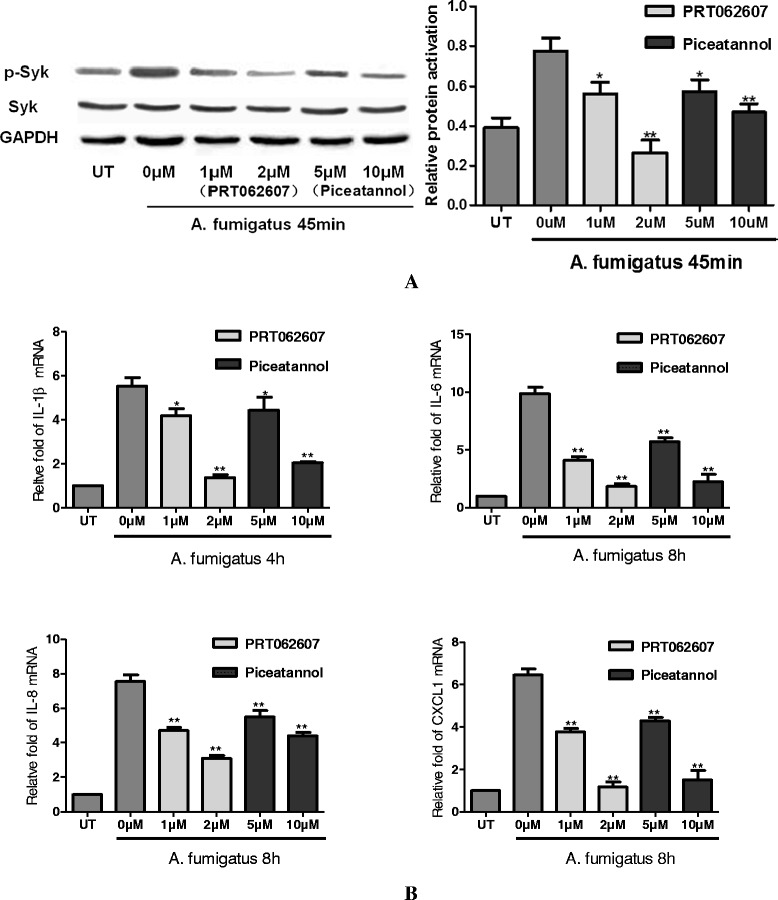
Change of protein and mRNA with or without the pretreatment of Syk inhibitors. After pretreated with PRT062607 and Piceatannol for 30 min before A. fumigatus hyphae stimulation, activation of Syk was inhibited by 1 μM PRT062607 (p < 0.05), 2 μM PRT062607 (p < 0.01), 5 μM Piceatannol (p < 0.05), 10 μM Piceatannol (p < 0.01) **a** compared with untreated cells. mRNA expression of IL-1β, IL-6, IL-8 and CXCL1 induced by A. fumigatus hyphae were significantly suppressed **b**. mRNA expression of IL-1β, IL-6, IL-8, CXCL1 downregulated significantly by 1 μM PRT062607, 2 μM PRT062607, 5 μM Piceatannol, 10 μM Piceatannol. **means p < 0.01, *p < 0.05

To further investigate whether Syk signaling pathway involved in A. fumigatus hyphae stimulated inflammatory response, THCEs were pretreated with Syk inhibitors PRT062607(1 and 2 μM) and Piceatannol (5 and 10 μM) for 30 min and stimulated with A. fumigatus hyphae for 45 min. From the results of Fig. 1 and Fig. 2, peak times were selected to test the effect of Syk inhibitors. As is shown in Fig. 3, after stimulation with A. fumigatus hyphae (5 × 10^6^/mL) for 45 min, activation of Syk and the mRNA expression of IL-6, IL-8, CXCL1 and IL-1β were significantly increased, which consistent with the above results. After pretreated with PRT062607 and Piceatannol for 30 min before A. fumigatus hyphae stimulation, activation of Syk was inhibited by 1 μM PRT062607 (p < 0.05), 2 μM PRT062607 (p < 0.01), 5 μM Piceatannol (p < 0.05), 10 μM Piceatannol (p < 0.01) (Fig. 3a) compared with untreated cells. mRNA expression of IL-6, IL-8, CXCL1 and IL-1β induced by A. fumigatus hyphae were significantly suppressed (Fig. 3b). mRNA expression of IL-1β (1 μM PRT062607 (p < 0.05), 2 μM PRT062607 (p < 0.01), 5 μM Piceatannol (p < 0.05), 10 μM Piceatannol (p < 0.01)), IL-6 (1 μM PRT062607 (p < 0.01), 2 μM PRT062607 (p < 0.01), 5 μM Piceatannol (p < 0.01), 10 μM Piceatannol (p < 0.01)), IL-8 (1 μM PRT062607 (p < 0.01), 2 μM PRT062607 (p < 0.01), 5 μM Piceatannol (p < 0.01), 10 μM Piceatannol (p < 0.01)), CXCL1 (1 μM PRT062607 (p < 0.01), 2 μM PRT062607 (p < 0.01), 5 μM Piceatannol (p < 0.01), 10 μM Piceatannol (p < 0.01)) were downregulated significantly. The results confirmed that Syk plays an important role in A. fumigatus hyphae induced inflammation in THCEs.

## Discussion

Fungal keratitis is a very common and serious infective corneal disease in many developing countries [16–18]. Corneal epithelium is an important biological barrier in the eye, and pathogenic fungi invade the cornea through injured epithelium, producing various enzymes and causing corneal ulcer [2].

Innate immune response against A. fumigatus plays a crucial role in controlling infection [19]. Inflammatory mediators participate in the development of fungal keratitis. In our study, we showed that A. fumigatus hyphae significantly upregulate the mRNA levels of inflammatory cytokines IL-1β, IL-6 and chemokines IL-8, CXCL1 by THCEs. Research has shown the increased IL-1β expression in mice pseudomonas aeruginosa keratitis, which can aggravate the inflammatory response and corneal tissue destruction [20]. IL-6 is an important inflammation factor and it plays defensive role in the corneal inflammation response [21]. Chemokines can mediate the recruitment of neutrophils and macrophages to eradicate pathogenic fungi. IL-8 is a chemokine in inflammatory response. It can aggregation the polymorphonuclear neutrophils (PMNs) in the immune process [22]. Study in LPS induced keratitis models of mice showed that the expression of CXCL1 increased and it plays an important role in the expression of chemokine and neutrophil infiltration [23–25]. This suggests that the corneal epithelial cells can express more IL-1β, IL-6, IL-8 and CXCL1 after identifying the fungus, which resist the fungal infection and induce inflammatory cells infiltration to remove pathogenic fungi.

The immune system plays the leading role through the cooperation of different PRRs. Studies have shown that many PRRs participate in the antifungal immune response system, such as TLR2, TLR4, Dectin-1, Dectin-2 and Mincle, etc [26–29]. Syk, as the core part in the downstream signaling pathway of many receptors, plays a key role in the innate immune system in response to fungal infection [30]. Syk pathway is the most important intracellular signaling pathways of Dectin-1. Studies had showed that Dectin-1 dependent cytokine production, MAPK activation and NF-κB activation can be inhibited by Syk deficiency or Syk inhibitors [31]. Dominikus Strasser etc. found that zymosan could stimulate the production of p-Syk in bone marrow-derived dendritic cells compared with the normal group [32]. Syk-related signal transduction mechanism is very complicated. Studies have shown that it activates the downstream molecule CARD9, which is the necessary signaling molecule that connect the coupling receptor and NF-κB pathway [33]. CARD9 can form a trimolecular complex with Bcl10 and MALT1 and they can activate the transcription factor NF-κB, causing production of inflammatory cytokines, such as IL-6, TNF-α and ProIL-1β [34]. In addition, Syk can also motivate the NLRP3 inflammatory complexes under the action of ROS generated by ERK, cause the activation of Caspase-1, and change the ProIL-1β into the activated form, IL-1β [9, 35, 36]. Our findings showed that A. fumigatus hyphae treated THCEs had higher p-Syk protein level compared with untreated THCEs. These results suggest that Syk involved in the innate immunity of the corneal resistance to fungal infection.

Inflammatory mediators stimulated by A. fumigatus hyphae were markedly blocked by Syk inhibitors in cultured THCEs, suggesting that Syk signaling pathways involved in the innate immune response of THCE cells against A. fumigatus hyphae. Syk activation is related to the expression of inflammatory mediators (IL-1β, IL-6, IL-8 and CXCL1). These findings demonstrated that inflammatory cytokines and chemokines production can through activation of Syk after A. fumigatus hyphae stimulation.

## Conclusion

In conclusion, our findings demonstrate that A. fumigatus hyphae stimulate the production of inflammatory mediators and Syk is activated after the fungal infection in the human corneal epithelial cells. We further prove that Syk inhibitors can suppressed the production of inflammatory mediators apparently. This study suggests that A. fumigatus hyphae stimulate the expression of inflammatory cytokines and chemokines through Syk signaling pathways in corneal epithelial cells. With the further study of Syk pathways will provide more targets for the prevention and treatment of fungal infections.

### Ethics statement

The research has been performed in accordance with the Declaration of Helsinki.

## References

[1] Nielsen E, Heegaard S, Prause JU, Ivarsen A, Mortensen KL, Hjortdal J. Fungal Keratitis – Improving Diagnostics by Confocal Microscopy. Case Rep Ophthalmol. 2013;4(3):303–10. 10.1159/000357558 PMC3901632 24474933

[2] Marakalala MJ, Kerrigan AM, Brown GD (2011). Dectin-1: a role in antifungal defense and consequences of genetic polymorphisms in humans. Mamm Genome.

[3] Kvarnhammar AM, Cardell LO (2012). Pattern-recognition receptors in human eosinophils. Immunology.

[4] Osorio F, Reis e Sousa C (2011). Myeloid C-type lectin receptors in pathogen recognition and host defense. Immunity.

[5] Kerrigan AM, Brown GD (2010). Syk-coupled C-type lectin receptors that mediate cellular activation via single tyrosine based activation motifs. Immunol Rev.

[6] Graham MT, Abram CL, Hu Y, Lowell CA. Expression of the TEL-Syk fusion protein in hematopoietic stem cells leads to rapidly fatal myelofibrosis in mice. PLoS One. 2013;8(10):e77542. 10.1371/journal.pone.0077542 PMC3792906 24116232

[7] Shakeel S, Mahjabeen I, Kayani MA, Faryal R. Association of SYK genetic variations with breast cancer pathogenesis. Asian Pac J Cancer Prev. 2013;14(5):3309–14. 10.7314/apjcp.2013.14.5.3309 23803121

[8] Dong SW, Zhang P, Zhong RR, Liu Q. Expression of putative tumor suppressor gene spleen tyrosine kinase in esophageal squamous cell carcinoma. Clin Lab. 2013;59(5–6):647–53. 10.7754/clin.lab.2012.120414 23865365

[9] Saïd-Sadier N, Padilla E, Langsley G, Ojcius DM. Aspergillus fumigatus stimulates the NLRP3 inflammasome through a pathway requiring ROS production and the Syk tyrosine kinase. PLoS One. 2010;5(4):e10008. 10.1371/journal.pone.0010008 PMC2848854 20368800

[10] Pitcher LA, van Oers NS (2003). T-cell receptor signal transmission: who gives an ITAM?. Trends Immunol.

[11] Kerrigan AM, Brown GD (2011). Syk-coupled C-type lectins in immunity. Trends Immunol.

[12] Ting LIU, Yuan-yuan XU, Hao CHEN, Li-xi XIE. Rabbit model of aspergillus keratitis induced by modified corneal surface lens method. Zhonghua Shiyan Yanke Zazhi. 2011;29(2):101–106.

[13] Spurgeon SE, Coffey G, Fletcher LB, Burke R, Tyner JW, Druker BJ, et al. The selective SYK inhibitor P505-15 (PRT062607) inhibits B cell signaling and function in vitro and in vivo and augments the activity of fludarabine in chronic lymphocytic leukemia. J Pharmacol Exp Ther. 2013;344(2):378–87. 10.1124/jpet.112.200832 PMC3558816 23220742

[14] Zheng Z, Li Z, Chen S, Pan J, Ma X. Tetramethylpyrazine attenuates TNF-α-induced iNOS expression in human endothelial cells: Involvement of Syk-mediated Activation of PI3K-IKK-IκB signaling pathways. Exp Cell Res. 2013;319(14):2145–51. 10.1016/j.yexcr.2013.05.018 23726836

[15] De Paiva CS, Corrales RM, Villarreal AL, Farley WJ, Li DQ, Stern ME, et al. Corticosteroid and doxycycline suppress MMP-9 and inflammatory cytokine expression, MAPK activation in the corneal epithelium in experimental dry eye. Exp Eye Res. 2006;83:526–35. 10.1016/j.exer.2006.02.004 16643899

[16] Pérez-Balbuena AL, Vanzzini-Rosano V, Valadéz-Virgen Jde J, Campos-Möller X. Fusarium keratitis in Mexico. Cornea. 2009;28:626–30. 10.1097/ICO.0b013e31819bc2ea 19512910

[17] Wang L, Sun S, Jing Y, Han L, Zhang H, Yue J. Spectrum of fungal keratitis in central China. Clin Experiment Ophthalmol. 2009;37:763–71. 10.1111/j.1442-9071.2009.02155.x 19878220

[18] Bharathi MJ, Ramakrishnan R, Meenakshi R, Padmavathy S, Shivakumar C, Srinivasan M. Microbial keratitis in South India: influence of risk factors, climate, and geographical variation. Ophthalmic Epidemiol. 2007;14:61–9. 10.1080/09286580601001347 17464852

[19] Balloy V, Chignard M (2009). The innate immune response to Aspergillus fumigatus. Microbes Infect.

[20] Rudner XL, Kernacki KA, Barrett RP, Hazlett LD. Prolonged elevation of IL-1 in Pseudomonas aeruginosa ocular infection regulates macrophage-inflammatory protein-2 production, polymorphonuclear neutrophil persistence, and corneal perforation. The Journal of Immunology. 2000;164(12):6576-82. 10.4049/jimmunol.164.12.6576 10843717

[21] Cole N, Bao S, Stapleton F, Thakur A, Husband AJ, Beagley KW, et al. Pseudomonasaeruginosa keratitis in IL-6-deficient mice. International archives of allergy and immunology.2003;130(2):165-172. 10.1159/000069006 12673071

[22] Kernacki KA, Barrett RP, Hobden JA, Hazlett LD. Macrophage inflammatory protein-2 is a mediator of polymorphonuclear neutrophil influx in ocular bacterial infection. The Journal of Immunology.2000;164(2):1037-45. 10.4049/jimmunol.164.2.1037 10623854

[23] Shao H, Scott SG, Nakata C, Hamad AR, Chakravarti S. Extracellular Matrix Protein Lumican Promotes Clearance and Resolution of Pseudomonas aeruginosa Keratitis in a Mouse Model. PLoS One. 2013;8:e54765. 10.1371/journal.pone.0054765 PMC3554612 23358433

[24] Lin M, Carlson E, Diaconu E, Pearlman E. CXCL1/KC and CXCL5/LIX are selectively produced by corneal fibroblasts and mediate neutrophil infiltration to the corneal stroma in LPS keratitis. J Leukoc Biol. 2007;81:786–92. 10.1189/jlb.0806502 PMC3909486 17110418

[25] Chintakuntlawar AV, Chodosh J (2009). Chemokine CXCL1/KC and its receptor CXCR2 are responsible for neutrophil chemotaxis in adenoviral keratitis. J Interferon Cytokine Res.

[26] Gersuk GM, Underhill DM, Zhu L, Marr KA. Dectin-1 and TLRs permit macrophages to distinguish between different Aspergillus fumigatus cellular states. J Immunol. 2006;176:3717–24. 10.4049/jimmunol.176.6.3717 16517740

[27] Hohl TM, Van Epps HL, Rivera A, Morgan LA, Chen PL, Feldmesser M, et al. Aspergillus fumigatus triggers inflammatory responses by stage-specific betaglucan display. PLoS Pathog. 2005;1:e30. 10.1371/journal.ppat.0010030 PMC1287910 16304610

[28] Bretz C, Gersuk G, Knoblaugh S, Chaudhary N, Randolph-Habecker J, Hackman RC, et al. MyD88 signaling contributes to early pulmonary responses to Aspergillus fumigatus. Infect Immun. 2008;76:952–8. 10.1128/IAI.00927-07 PMC2258799 18039832

[29] Werner JL, Metz AE, Horn D, Schoeb TR, Hewitt MM, Schwiebert LM, et al. Requisite role for the dectin-1 beta-glucan receptor in pulmonary defense against Aspergillus fumigatus. J Immunol. 2009;182:4938–46. 10.4049/jimmunol.0804250 PMC3434356 19342673

[30] Robinson MJ, Osorio F, Rosas M, Freitas RP, Schweighoffer E, Gross O, et al. Dectin-2 is a Syk-coupled pattern recognition receptor crucial for Th17 responses to fungal infection. J Exp Med. 2009;206(9):2037–51. 10.1084/jem.20082818 PMC2737172 19703985

[31] Drummond RA, Saijo S, Iwakura Y, Brown GD. The role of Syk/CARD9 coupled C-type lectins in antifungal immunity. Eur J Immunol. 2011;1(2):276–81. 10.1002/eji.201041252 PMC3434674 21267996

[32] Strasser D, Neumann K, Bergmann H, Marakalala MJ, Guler R, Rojowska A, et al. Syk kinase-coupled C-type lectin receptors engage protein kinase C-σ to elicit Card9 adaptor-mediated innate immunity. Immunity. 2012;36(1):32–42. 10.1016/j.immuni.2011.11.015 PMC3477316 22265677

[33] Hara H, Ishihara C, Takeuchi A, Imanishi T, Xue L, Morris SW, et al. The adaptor protein CARD9 is essential for the activation of myeloid cells through ITAM-associated and Toll-like receptors. Nat Immunol. 2007;8(6):619–29. 10.1038/ni1466 17486093

[34] Gross O, Gewies A, Finger K, Schäfer M, Sparwasser T, Peschel C, et al. Card9 controls a non-TLR signaling pathway for innate anti-fungal immunity. Nature. 2006;442(7103):651–6. 10.1038/nature04926 16862125

[35] Kumar H, Kumagai Y, Tsuchida T, Koenig PA, Satoh T, Guo Z, et al. Involvement of the NLRP3 inflammasome in innate and humoral adaptive immune responses to fungal beta-glucan. J Immunol. 2009;183(12):8061–7. 10.4049/jimmunol.0902477 20007575

[36] Gross O, Poeck H, Bscheider M, Dostert C, Hannesschläger N, Endres S, et al. Syk kinase signalling couples to the Nlrp3 inflammasome for anti-fungal host defence. Nature. 2009;459(7245):433–6. 10.1038/nature07965 19339971

